# mzRAPP: a tool for reliability assessment of data pre-processing in non-targeted metabolomics

**DOI:** 10.1093/bioinformatics/btab231

**Published:** 2021-04-07

**Authors:** Yasin El Abiead, Maximilian Milford, Reza M Salek, Gunda Koellensperger

**Affiliations:** Department of Analytical Chemistry, University of Vienna, Vienna 1090, Austria; Department of Analytical Chemistry, University of Vienna, Vienna 1090, Austria; International Agency for Research on Cancer, Section of Nutrition and Metabolism, Lyon 69008, France; Department of Analytical Chemistry, University of Vienna, Vienna 1090, Austria

## Abstract

**Summary:**

Reliability assessment of automated pre-processing of liquid chromatography-high resolution mass spectrometry data presents a significant challenge. Here, we present a tool named mzRAPP, which generates and validates a benchmark from user-supplied information and later utilizes it for reliability assessment of data pre-processing. As a result, mzRAPP produces several performance metrics for different steps of the pre-processing workflow, supporting five of the most commonly used pre-processing tools.

**Availability and implementation:**

mzRAPP is implemented in R and can be downloaded from GitHub under GNU GPL v.3.0 licence. Extensive documentation, background and examples are available at (https://github.com/YasinEl/mzRAPP).

**Supplementary information:**

Supplementary data are available at *Bioinformatics* online.

## 1 Introduction

**Fig. 1.**
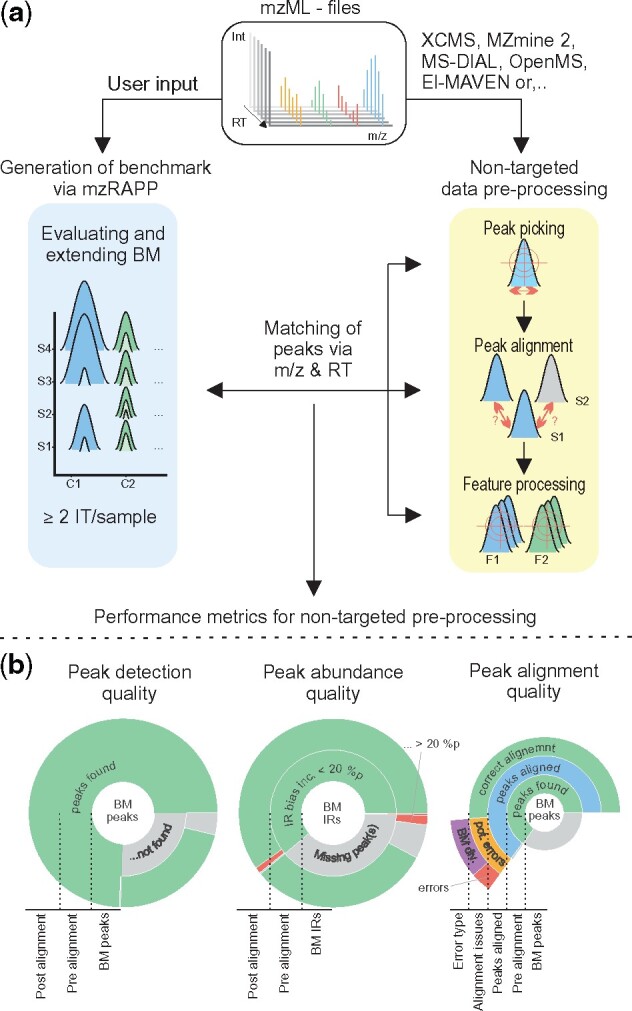
Overview of the mzRAPP workflow. (**a**) The mzML files are analyzed and inspected via non-targeted data pre-processing (e.g. via XCMS, MZmine 2, MS-DIAL) and then used to generate and validate a BM using known IT ratios. The generated BM is then utilized to assess the performance of NPP peak picking, peak alignment and feature processing across NPP methods. Subsequently, different performance metrics can be derived as a result. (**b**) The mzRAPP output graphics include different sunburst quality evaluation plots. From left to right, sunburst plots describe the peak detection (true positive/false negative peaks) and abundance (IT ratio bias) quality before (inner donut) and after alignment (outer donut). The right sunburst plot visualizes errors that occurred during the alignment step. More details are provided in Supplementary Material S2

To date, several different software solutions for data pre-processing have been proposed, and new ones are published continuously. However, reproducibility, comparability and reliability of results generated via such algorithms remain challenging and difficult to assess or compare. Difficulties in the evaluation are primarily due to complex parameter options used for pre-processing within various tools and the complexity of the analysis, which can potentially affect and vary the outcome (Li *et al.*, 2018). In metabolomics and particularly for non-targeted metabolomics, there are no ‘gold standard’ experimental datasets or a trusted set of methods to evaluate parameter choices and tools [e.g. Sanger sequencing for genomics (Sanger *et al.*, 1977)]. Moreover, differences in instrumentation, acquisition strategies or sample complexity might not draw a general conclusion from a potential gold standard dataset. While there are software packages for automatized non-targeted data pre-processing (NPP) parameter optimization and adaption [e.g. IPO (Libiseller et al., 2015), Autotuner (McLean *et al.*, 2020)], they are not meant to assess the general quality of NPP results (More details in Supplementary Material S1). Hence there is a need for a strategy to assess the reliability of non-targeted data pre-processing (NPP) for LC-HRMS data on a routine case by case basis. This led us to develop the R package mzRAPP (Reliability Assessment for NPP), which enabled the extraction of well-defined NPP performance metrics from existing experimental datasets, by generating a benchmark (BM) subset to evaluate NPP-results (Fig. 1). For benchmarking, mzRAPP relies on user-provided input, specific to the experimental chromatographic setup [approximated retention time (RT) boundaries for a subset of compounds with known molecular formulas] for the generation of a BM. This BM is then used as a reference point for deriving performance metrics for NPP conducted on the same raw data. The reliability of NPP performance metrics is ensured by considering a wide range of orthogonal information. Broad applicability in the interdisciplinary field of metabolomics is enabled via graphical user interface (GUI) and support of more than five high cited NPP tools.

## 2 Implementation and methods

The mzRAPP-package has been developed with the R environment (version 4.0.3) and implemented as a shiny app for ease of use. It can be navigated in a two-step procedure, resulting in ten different NPP performance metrics. The first step is a module responsible for confirming the consistency of user-provided information (RT boundaries for different molecular formulas) with information extracted from the raw data in order to generate a sub-dataset-specific BM. This is achieved by comparing the enviPat-predicted isotopologue (IT) ratios (Loos *et al.*, 2015) to those extracted from mzML files using user-supplied boundaries (see GitHub for details). Next, the BM is used as a reference point leading to the generation of several NPP-performance metrics for the evaluation of chromatographic peak picking and alignment as well as the aligned features, respectively. The output metrics include measurements to judge the proportion of missed peaks, quality of reported peak abundances, nature of missed peaks and alignment error counts. Interactive plots providing an overview and context to all metrics can be inspected interactively within the shiny app. As a result, several different NPP algorithms across tools with different parameter choices, can be evaluated for an optimal outcome. Below are the main theme and underlying methods developed for the mzRAPP package and the description of important key functionalities.

### 2.1 Main theme

The BM construction relied on the integration of key functions from enviPat (the IT pattern prediction from molecular formulas), XCMS (Tautenhahn *et al.*, 2008), MSnbase (Gatto and Lilley, 2012) (efficient extraction and structuring of raw data) and MetaClean (Chetnik *et al.*, 2020) (extraction of different peak variables). Data table operations were conducted via functionalities from the data.table package. Parallel processing capability has been implemented in mzRAPP via future package (Bengtsson, 2020). GUI and plots are adopted via shiny and ggplot2/plotly, respectively.

### 2.2 Key functions

#### 2.2.1 BM generation

BM generation was facilitated via a cascade of three functions performing IT pattern prediction (*get_mz_table*), extraction of chromatogram information (*get_ROIs*) and ultimately peak extraction, consistency checks and filtering (*find_bench_peaks*). Each function returns a *data.table* object serving as argument for the subsequent proceeding functions. In doing so mzRAPP relies on functionalities from enviPat, XCMS (*xcmsRaw* and *findmzROI*) and MSnbase (*readMSData* and *chromatogram*) architectures. The generated BM can then be used for NPP reliability assessment as described below. More details are provided in Supplementary Material S2.

#### 2.2.2 Assessing NPP reliability

Since the BM can be edited by the user its data structure is checked via *check_benchmark_input* before being used for NPP assessment. The mzRAPP-package supports output formats from the five most common NPP tools, such as XCMS, MS-DIAL (Tsugawa *et al.*, 2015) and MZmine 2 (Pluskal *et al.*, 2010). The *check_nonTargeted_input* function checks the presence of all components necessary for subsequent matching procedures and generates a generic format output for downstream processing. Next mzRAPPs *compare_peaks* function performs a series of non-equivalent joins between the BM and supplied NPP output tables. The result is returned as a *list* object which serves as input for different downstream analysis and plotting functions [e.g. *derive_performance_metrics* (details are provided in Supplementary Material S2) and *plot_sunburst_peakQuality*]. Elements of the list are themed around conducted joins or classifications, details of which can be viewed using function-help. Output metrics include measurements to judge the proportion of missed peaks, quality of peak abundances, nature of missed peaks and the alignment error counts. Empirical confidence intervals (alpha = 0.95) for all metrics are estimated by bootstrapping (as employed in the boot package) for the supplied molecules.

#### 2.2.3 Graphical user interface

A comprehensive GUI was developed to help metabolomics scientists with different computational skill sets access mzRAPP software solutions for NPP evaluation. The *callmzRAPP* function allows users to generate BMs and assess NPP without requiring any command-line functions.

## Supplementary Material

btab231_Supplementary_Data
