# Supplementary material for: mzRAPP: a tool for reliability assessment of data pre-processing in non-targeted metabolomics
Source: Bioinformatics. 2021 Apr 7;37(20):3678–80. doi: 10.1093/bioinformatics/btab231 (PMC8545297; doi:10.1093/bioinformatics/btab231)
Supplement: btab231_Supplementary_Data [file btab231_supplementary_data.pdf]

Supplementary material

Supplementary Information 1:

Several useful non-targeted data pre-processing parameter optimization tools are available (e.g., IPO, Auto-Tuner, MetaboAnalystR 3.0,..). They base their optimization on the general existence of isotopologues of unknown identity (IPO and MetaboAnalystR 3.0) or characteristics of unverified extracted ion chromatograms (Auto-Tuner). Since these are universal properties of liquid chromatography-high resolution mass spectrometry data, all of the tools above apply to almost any dataset. However, none of these strategies enable checking the recovery of known peaks and their representation in the NPP-output (e.g., the correctness of peak alignment and reported peak areas). The mzRAPP-package closes these gaps by relying on user-provided (automatically validated) information on molecules with known identity, which can be used as ground-truth. It is worth noting that mzRAPP's purpose is to report NPP-performance metrics to the user. While this information can be used to adapt NPP parameters, mzRAPP is not primarily intended for parameter optimization. Instead, it allows users to make informed decisions on how to proceed with their NPP-results.

Supplementary Information 2:

Here we briefly summarize the principles behind the benchmark generation and main-performance metrics. Additional details (and future updates) are provided in the mzRAPP Readme (https://github.com/YasinEl/mzRAPP).

Benchmark generation: mzRAPP takes manually inspected peak lists (including retention time boundaries) of molecules with known molecular formulas as an input. Peaks of all predicted isotopologues are then automatically extracted from mzML files. All peaks for which a minimum of two isotopologues (matched to the predicted pattern in accurate mass, peak height and area; matched to each other via peak shape correlation) are found are added to the benchmark peak list (BM).

Found/Not found peaks: To calculate the number of BM peaks not found via non-targeted data pre-processing (NPP), the number of all BM peaks is subtracted by the number of BM peaks for which no match has been found. This is done before and after peak alignment.

Missed peaks: Often missing values have to be imputed for statistical evaluations. For each not-found Peak, mzRAPP evaluates whether it led to a missing value higher than 1.5 times the abundance of the lowest NPP-found peak in the corresponding BM feature.

Peak abundance: To estimate the quality of NPP-reported peak abundances, isotopologue ratios (IRs) calculated from BM peak areas (confirmed via IRs predicted from molecular formulas) are compared to IRs calculated from NPP-reported peak areas. If a given NPP-IR bias is more than 20 percentage points (%p) higher than in the respective BM-IR, it is flagged.

Peak alignment: Peak alignment is responsible for associating peaks in different samples with each other. mzRAPP counts the number of times isotopologues of the same molecular compound are aligned in different ways within the same sample as errors. For more details and other alignment flags identifiable by mzRAPP, please check the Readme.
